# Typification of binomials in Xyris section Nematopus (Xyridaceae) published by L.A. Nilsson

**DOI:** 10.3897/phytokeys.80.12348

**Published:** 2017-06-05

**Authors:** Maria das Graças Lapa Wanderley

**Affiliations:** 1 Instituto de Botanica, São Paulo, Brazil

**Keywords:** *Xyris*, Xyridaceae, lectotype, holotype, nomenclature

## Abstract

A nomenclatural revision of fifteen taxa of *Xyris* (Xyridaceae) described by L.A. Nilsson (1892) is presented as part of a taxonomic revision of the genus in Brazil. All the protologues and type collections of these taxa were studied. The type collections were examined in the respective herbarium collections where they are preserved and complemented by images available on herbarium websites and from JSTOR Global Plants. Lectotypes were selected for *Xyris cristata* L.A.Nilsson, *X. glaziowii* L.A.Nilsson and *X. insignis* L.A.Nilsson. The holotypes, of *X. glandacea* L.A.Nilsson and *X. stenophylla* L.A.Nilsson, were discovered at the herbaria of Uppsala (UPS) and Berlin (B) respectively, and provided with their correct determinations.

## Abstract (nl)

É apresentada a revisão nomenclatural de quinze taxa do gênero *Xyris* (Xyridaceae) descritos por L.A. Nilsson (1892), como parte da revisão taxonômica do gênero no Brasil. Foram analisados os protólogos destes taxa e as coleções-tipo nos acervos dos respectivos herbários onde os mesmos estão preservados e complementarmente por imagens disponíveis nos websites dos herbários e por meio do JSTOR Global Plants. Foram designados os lectótipos para *Xyris cristata* L.A.Nilsson; *X. glaziowii* L.A.Nilsson and *X. insignis* L.A.Nilsson. Dois holótipos com indicações equivocadas foram aqui encontrados e corretamente determinados: *Xyris glandacea* L.A.Nilsson, no Herbário UPS e *X. stenophylla* L.A.Nilsson, em B.

## Introduction

The family Xyridaceae comprises about 400 species (Wanderley 2011, Silva and Wanderley 2013, Mota and Wanderley 2014, Santos-Guedes and Wanderley 2015), of which approximately 92% occur in Brazil (Flora do Brasil 2016). The most recent monograph of Xyridaceae in Brazil (Smith and Downs 1968) recognized 115 species of *Xyris*. However, a large number of Brazilian species have been described since (Kral and Smith 1980, 1982, Wanderley 1983, 1986, Wanderley and Cerati 1987, Kral and Wanderley 1988a, 1988b, 1993, Wanderley 2003, 2010, Mota and Wanderley 2013, Mota and Wanderley 2014, Wanderley and Mota 2015, Lozano et al. 2016), increasing the number of taxa by approximately 44% over the last three decades.

While undertaking taxonomic studies of Brazilian Xyridaceae, it was discovered that a number of species of the genus *Xyris* needed nomenclatural revision. Although Smith and Downs (1968), proposed typifications of many taxa of *Xyris* as part of their treatment, many others still do not have any indication of typification, or there are problems to be solved. In some cases, unrecognized type material was found in the general collections of herbaria with the consequence that these types are also not available on herbaria websites or by JSTOR Global Plants (Ithaka 2015).

In this paper, we revised the typification of fifteen Brazilian taxa described by L.A. Nilsson (1892), most of whose type collections are housed at the herbaria of two research institutions, the Swedish Museum of Natural History (S) and Uppsala University Museum of Evolution, Botany Section (UPS). Both herbaria were the institutions where the author worked during the 19th century. In his treatment, “Studien über die Xyrideen”, Nilsson (1892) studied 109 taxa of *Xyris*, of which 53 belong to section Nematopus. Two were placed in a “dubia” section by the author, i.e. without sectional affiliation, but these two taxa are assigned here to Xyris section Nematopus. Following a recent visit to the two Swedish herbaria, fifteen names were reviewed and typified, of which twelve are accepted names and three are synonyms, all included in Xyris section Nematopus. Lectotypes are proposed here for three names, chosen in accordance with the original descriptions of the taxon, so as to enhance nomenclatural stability and following the author’s original intentions. In the case of a previous intention of indication by another author, the priority of designation was accepted, according to the International Code of Nomenclature for Algae, Fungi and Plants (McNeill et al. 2012), unless the designation was incorrect, and bearing in mind if the indicated material is representative of the species (Prado et al. 2015). Based on the review of all the protologues and type collections of the studied taxa, lectotypes are designated for *Xyris cristata* L.A. Nilsson, *X. glaziowii* L.A. Nilsson and *X. insignis* L.A. Nilsson. Two holotypes with equivocal determinations are reported and corrected here: *Xyris glandacea* L.A. Nilsson, housed at the Uppsala herbarium (UPS) and *X. stenophylla* L.A. Nilsson, at Berlin (B), both designated equivocally as lectotypes earlier by Smith and Downs (1968). Some types cited by Smith and Downs (1968) simply as “type”, were updated to lectotype status, and isotypes and isolectotypes are also reported here.

## Material and methods

In this study, we analysed the protologues and morphological features from specimens of the following herbaria (* visited herbaria): B*, BR, K*, M*, NY*, P*, S*, US* and UPS* (see Thiers 2016 for acronyms). Typifications were carried out according to the current edition of the International Code of Nomenclature for Algae, Fungi and Plants (McNeill et al. 2012), as well as recommendations by Prado et al. (2015). The accepted names of the taxa are shown in bold italics, and the non-accepted ones only in italics. Images were studied using a source from the herbaria B, BR and M or by using digital images available on the JSTOR Global Plants website (Ithaka 2015). All the original protologues were reviewed. Citations of type specimens are given as follows; when labelled by barcode, this is cited immediately after the herbarium acronym; if there is no barcode the herbarium registration number is cited, as in the case of the S herbarium; when the exsicatae have no registration numbers, as in the case of the UPS herbarium, just the respective herbarium acronym is cited.

## Typifications

### 1. Xyris cristata

Taxon classificationPlantaePoalesXyridaceae

L.A.Nilsson, Kongl. Svenska Vetenskap.-Akad. Handl. 24(14): 56. 1892.

#### Type.

“Brasilien”. Bahia “in paludosis ad Itahype flum. Com. Dos Ilheos”, *Martius s.n.* (lectotype, **designated here**: M! *pro parte*: plant marked as M0241131). Other: “Brasilien”. Minas Gerais, Vila do Príncipe, “in editis camporum humidis prope V. do Principe”, *Martius s.n.* (syntype: M! *pro parte*: plant marked as M0241116).

=***Xyris minarum*** Seub. in Martius, Fl. Bras. 3(1): 215. 1855.

This species is a synonym of *X. minarum* Seub. Nilsson (1892) cited two syntypes for *Xyris cristata* in the protologue, both collected by Martius: 1) *Martius s.n.*, from Minas Gerais, Vila do Príncipe, (“in editis camporum humidis prope V. do Principe”), and 2) *Martius s.n.* from Bahia, “in paludosis ad Itahype flum. Com. Dos Ilheos”. However, both collections contain a mixture of species. The sheet from Bahia presents a mixture of *Xyris cristata* and *X. rupicola* Kunth, presumably due to their similarity in habit, a common problem in identification of *Xyris* species. On this sheet, the plants of the two species are marked with different barcodes, *X. rupicola* (M0241130) and *X. cristata* (M0241131). The specimen of *X. cristata* is positioned in the center of the sheet marked with the number “2”, whereas the two specimens positioned laterally and marked “1” correspond to *X. rupicola*. The material (M0241131) agrees with the description of the species provided in the protologue.

The collection from Minas Gerais includes the other syntype of *Xyris cristata*, but also plants of a third species, *X. subsetigera* Malme. The specimens of each species mixed together in this Martius collection agree with their respective protologues and both names are validly published. The mixed collection has been separated into three exsiccatae; the plants of *X. subsetigera* are now registered as M0241136 and M0241137, whereas that of *X. cristata* is registered as M0241116. This latter plant is a poor sample of *X. cristata*, represented only by three peduncles and three spikes.

After studying the two syntypes (M0241131, M0241116), the Bahian collection was selected as the lectotype of *Xyris cristata*, because it is a more complete specimen.

### 2. Xyris filifolia

Taxon classificationPlantaePoalesXyridaceae

L.A.Nilsson, Kongl. Svenska Vetenskap.-Akad. Handl. 24(14): 43-44. 1892.

#### Type.

“Brasilien”. Minas Gerais, Caldas, *Regnell III 2051*, “in palude inter gramineas altas” (lectotype, designated by Smith & Downs, 1968: S! [S-R-6569]; isolectotypes: UPS!, US! [US00088150]).

In the protologue of the species, two collections were cited: *Regnell III 2051* and *Lindberg s.n.* The choice of lectotype was previously made by Smith and Downs (1968), who indicated the sheet *Regnell III 2051*, 30[?]-II-1880 as the “type”, deposited in S (S-R-6569), which by this indication became the lectotype. It is likely that during the distribution of the duplicates between the S (30-II-1880) and UPS (20-II-1880) herbaria, a change in the date was made in the transcription of the label. A second sheet is deposited in UPS (*Regnell III 2051*), although under a different date (13-II-1880), but there is no doubt that this material is part of the same collection (*Regnell III 2051*). This sheet from UPS (*Regnell III 2051*, 13-II-1880) contains a short description of the species (in handwriting similar to that of Nilsson) and the observation “in palude inter gramineas altas”, which occurs in the protologue. The Lindberg collection 556 (XII-1854), deposited in S (S-R-6570) and also cited in the protologue, is a syntype. This material comes from Minas Gerais, Caldas, and has the same location as the lectotype.

### 3. Xyris fusca

Taxon classificationPlantaePoalesXyridaceae

L.A.Nilsson, Kongl. Svenska Vetenskap.-Akad. Handl. 24(14): 57-58. 1892.

#### Type.

“Brasilien”. *Glaziou* 6747 (lectotype, designated as type by Smith & Downs, 1968: B! [B10 0243260]; isolectotypes: P! [P01679919], K! [K000587085]).

The Glaziou 6747 specimen from Brazil has three duplicates, of which B10 0243260 is the lectotype, designated as “type” by Smith and Downs (1968). The isolectotypes deposited in P and K are: P01679919, K000587085. The label of the sheet at P gives the origin of the collection as Brazil, Rio de Janeiro, Itatiaia, January 22, 1873. In the K isolectotype, there is no information on locality or date. The information given is the collector and the respective collection number, the origin as Rio de Janeiro, and the date as February 1874. The later date of the Kew specimen might signify when the specimen was received at K as a duplicate from P. However, there is no doubt that the three exsiccates are from the Glaziou 6747 collection.

### 4. Xyris glandacea

Taxon classificationPlantaePoalesXyridaceae

L.A.Nilsson, Kongl. Svenska Vetenskap.-Akad. Handl. 24(14): 50. 1892.

#### Type.

“Brasilien”. Minas Gerais, Caldas, *Regnel III 2065* (in herb. UPS) (holotype: UPS!; isotype: S! [S-R-6575]).

In his *Xyris glandacea* protologue, Nilsson (1892) cited the *Regnell III 2065* collection, and indicated UPS as the herbarium where it was deposited. However, Smith and Downs (1968), apparently ignoring the holotype, made an improper designation, and chose the isotype at S as the lectotype. The present study confirmed the presence of the holotype at UPS (*Regnell III 2065*), and that the duplicate housed at S is indeed the isotype. The holotype contains a short manuscript description that agrees with the species diagnosis. There is an attached envelope containing fragments of the inflorescence, confirming that it was used to describe the species. Both the holotype and isotype are compatible with the diagnosis and provide decisive confirmation of the rediscovery in the field of *X. glandacea* after a century, previously reported by Wanderley (2011). Due to earlier poor understanding of its identity, the name has been applied to various different species of *Xyris* in herbarium collections.

### 5. Xyris glaziowii

Taxon classificationPlantaePoalesXyridaceae

L.A.Nilsson, Kongl. Svenska Vetenskap.-Akad. Handl. 24(14): 61-62. 1892.

#### Type.

“Brasilien”, *Glaziou 8004* (B†; lectotype, **designated here**: P! [P00753734]; isolectotypes: NY! [NY00246945], BR [BR000000694356, scan!], P! [P00753735]).

= ***Xyris augusto-coburgii*** Szyszyl. ex G.Beck., Itin. Princ. S. Coburgii 2: 94. 1888.

This name is a synonym of *Xyris augusto-coburgii* Szyszyl. ex G.Beck. Although cited in the protologue as deposited in B, the type material of *X. glaziowii* is no longer to be found there. Dr. Robert Vogt, curator of B, has confirmed that the type material of *Xyris glaziowii* probably disappeared as a result of World War II damage to the B herbarium. Duplicates of *Glaziou 8004* at P, BR, NY, and a photograph at F were therefore examined with a view to selection of a lectotype. Smith and Downs (1968) indicated material deposited at P as the type. However, there are two sheets of *Glaziou 8004* in this herbarium, and the sheet numbered P00753734 was chosen and designated here as the lectotype, because of its more complete label data, stating that the material is from São Paulo, Fazenda da Bocaina, near Cascatinha do rio Mambucabo, collected on February 11, 1876. The isolectotypes are: P00753735, NY00246945 and BR000000694356, which although from the same collection (*Glaziou 8004*), have incomplete label information. The BR sheet indicates that it originated from Rio de Janeiro, while the label data of the NY sheet indicates its origin as São Paulo, Bocaina, as in the lectotype.

### 6. Xyris insignis

Taxon classificationPlantaePoalesXyridaceae

L.A.Nilsson, Kongl. Svenska Vetenskap.-Akad. Handl. 24(14): 44. 1892.

#### Type.

“Brasilien”. Minas Gerais, “in campis alpestribus ad Serro Frio”, *Martius s.n.* (in herb. Monac.) (lectotype, **designated here**: M! [M0241098]; isolectotype: M! [M0241097]).

According to the protologue, the Martius’s type collection is from Serro, Minas Gerais state, Brazil, but with no information of the collector number and date. It is deposited at the Munich herbarium (M) where there are two sheets of the same collection (M0241097 and M0241098). According to Smith and Downs (1968), the *Martius* collection was cited as a type, but without choosing one of the two *Maritus* sheets deposited in M. The lectotype is therefore designated here as the sheet M0241098. This choice was made because the material is more complete, corresponds to the original description of the species, and has annotations in pencil indicating that it is the holotype. Another indication of having been used by Nilsson (1892)to describe the species is the fact that there is an envelope with fragments of the dried floral spike. The duplicate sheet (M0241097) thus becomes an isolectotype.

### 7. Xyris laevigata

Taxon classificationPlantaePoalesXyridaceae

L.A.Nilsson, Kongl. Svenska Vetenskap.-Akad. Handl. 24(14): 50. 1892.

#### Type.

“Brasilien”. Rio de Janeiro, *Glaziou 1327* (lectotype, designated by Smith andDowns, 1968: B! [B100242250]; isolectotype: K! [K000587092]).

According to the protologue, the *Glaziou 13277* collection was used to describe the species, but without indication of the depository herbarium where it was deposited. Considering the intention of Smith and Downs (1968) in indicating the Berlin sheet (B100242250) as a “type”, their choice has priority and this sheet is therefore considered the lectotype. An isolectotype is deposited at K (K000587092).

### 8. Xyris longiscapa

Taxon classificationPlantaePoalesXyridaceae

L.A.Nilsson, Kongl. Svenska Vetenskap.-Akad. Handl. 24(14): 59-60. 1892.

#### Type.

“Brasilien”. Minas Gerais, “in palustribus Serra do Caraca”, *Martius s.n.* (holotype: M! [M0241109]).

In the protologue Nilsson (1892) indicated only the *Martius* collection without collector number, collected in Brazil, Minas Gerais state, in the Serra do Caraça, and deposited at the Munich herbarium (M). Since this collection consists of a unique sheet, it is consequently the holotype. The material is made up of three leaf rosettes, representative of the species and compatible with the protologue.

### 9. Xyris nigricans

Taxon classificationPlantaePoalesXyridaceae

L.A.Nilsson, Kongl. Svenska Vetenskap.-Akad. Handl. 24(14): 60. 1892.

#### Type.

“Brasilien”. Rio de Janeiro, *Glaziou 15513* (lectotype, designated by Smith & Downs, 1968: P! [P00752388]); isolectotypes: P! [P00752436]), K! [K000587098]), BR [BR000000694390, scan!]. Other: *Sellow s.n.* (syntype: B! [B 10 0242237]).

We examined two syntype collections of *X. nigricans*. Both are of good quality: 1) *Glaziou 15513*, has two sheets at P (P00752388; P00752436), one at K (K000587098) and one at BR (BR000000694390); 2) *Sellow s.n.* has a single sheet at B (B 10 0242237). The lectotype is confirmed as sheet P00752388 of the *Glaziou 15513* collection, previously indicated by Smith and Downs (1968) as the “type”. The label data of the lectotype states that it was collected in the state of Minas Gerais, at Alto de Itacolomy (“Haut Itacolomy”). The other three sheets at P (P 00752436), BR (BR000000694390) and K (K000587098) are thus isolectotypes. Although the species is endemic to Minas Gerais, equivocal information is given on the labels of the isolectotypes at K and BR, referring to the surroundings from Rio de Janeiro and Ouro Preto. However, there is no doubt that all of them are duplicates and part of the same collection *Glaziou 15513*, and each sheet was probably labelled by Glaziou. The exsiccate *Sellow s.n.*, deposited at B (B 10 0242237), is a syntype of *Xyris nigricans* and is compatible with the protologue.

### 10. Xyris obtusiuscula

Taxon classificationPlantaePoalesXyridaceae

L.A.Nilsson, Kongl. Svenska Vetenskap.-Akad. Handl. 24(14): 47. 1892.

#### Type.

“Brasilien”, *Sellow s.n.* (“in herb. Berol.”) (holotype: B! [B10 0242234]).

Nilsson (1892) cited the *Sellow s.n.* collection in the protologue as at the Berlin herbarium (B). Smith and Downs (1968) indicated the B sheet as the type, but without specifying the type’s category. Considering that only this sheet of this collection is known at B (B10 0242234) and Nilsson’s citation of B in the protologue, we can confirm that it is the holotype. Although no mention was made in the protologue of Sellow’s collecting number, the label states that it is *Sellow 1087*. The holotype is a poor specimen, consisting of only one plant with two incomplete spikes, together with added fragments of the inflorescence, but nevertheless corresponds to the characteristics of the species.

### 11. Xyris regnellii

Taxon classificationPlantaePoalesXyridaceae

L.A.Nilsson, Kongl. Svenska Vetenskap.-Akad. Handl. 24(14): 43. 1892.

#### Type.

“Brasilien”, Minas Gerais, Caldas, *Regnell III 2050* (lectotype, designated by Smith & Downs, 1968: S! [S-R-6609]; isolectotype: UPS!, *pro parte* P! [P01676114]). Other: “Brasilien”, Minas Gerais, Caldas, *Regnell III 2050* “loco paludoso in consortione graminearum excelsiorum” (syntype: UPS!).

In the protologue of the species, Nilsson (1892) cited two different collections from the same location (Brazil, Minas Gerais, Caldas) under the same number *Regnell III 2050*. The two collections can be recognized by the annotations referred to in the protologue: 1) “In palude inter gramineas alta”, and 2) “loco paludoso in consortione graminearum excelsiorum”. The sheets examined in the present study at P, S and UPS have different dates on their labels for this same collection - no dates are mentioned in the protologue. This variation of dates and other label information on the duplicate sheets of the type collection led to a more detailed analysis of the typification of this name, involving visits to both Swedish herbaria (UPS and S) and P, where the duplicates of the *Regnell III 2050* are deposited. Smith and Downs (1968) previously indicated as lectotype the sheet deposited in S (S-R-6609), and since this is representative of the species and protologue, it is confirmed here as the lectotype. It can also be recognized by the citation in the protologue (“Brasilien”, Minas Gerais, Caldas, *Regnell III 2050*); its label includes the date 10-01-1880. The isolectotype is deposited in UPS (*Regnell III 2050*) and has the same date as the lectotype (10-I-1880), includes a short description of the species (in pencil with similar handwriting to that of Nilsson), and the ecological description “in palude inter gramineas alta”, as cited in the protologue. Although the latter information does not appear on the lectotype sheet at S, there is no doubt that they are duplicates and that they complement each other in relation to the protologue. The syntype at UPS corresponds to the second material mentioned in the *Regnell III 2050* protologue (“loco paludoso in consortione graminearum excelsiorum”), and this sheet includes two dates: 20-I-1880 and 17-2-1880. It also has a brief description of the species and fragments of material in envelopes, indicating that they were examined by Nilsson when he prepared his original description. This material is thus very informative and complementary for interpreting this species name.

The P01676114 collection at Paris (P) is part of the type collection of *Xyris regnellii* (*Regnell III 2050*), and can be considered as a syntype, but only in part (*pro parte*), as it consists of a mixture of specimens of *Xyris regnellii* and *X. filifolia*. This sheet does not have a date of collection or any of the other information mentioned above, showing only the collector’s name and number. It is, however, the only reference to *X. regnellii* currently available on the JSTOR Global Plants (Ithaka 2015) website for type materials. Another specimen at P (P01727365), is possibly part of the type collection of *Xyris regnellii*, but as the number of the *Regnell* collection has not been recorded, it has not been included here in the above citation of duplicates of the type collection. Yet another P specimen, P01676128, which is of *X. regnellii*, was not considered for typification of the name, because it is labelled as *X. filifolia* and is a different collection (*Regnell III 2051*). The interpretation of *Xyris regnellii* and *X. filifolia* type material, described by Nilsson (1892) in the same work, was made more difficult as they were collected by Regnell in the same location (Minas Gerais, Caldas), and received consecutive numbers: *Regnell III 2050* for *Xyris regnellii*, and *Regnell III 2051* for *X. filifolia*. The mixture of material of these two species in the duplicates at P and the exchange of labels between them must have occurred during the original distribution of duplicates.

### 12. Xyris seubertii

Taxon classificationPlantaePoalesXyridaceae

L.A.Nilsson, Kongl. Svenska Vetenskap.-Akad. Handl. 24(14): 51. 1892.

#### Type.

Guyana: “British Guiana”, *Rich. Schomburgk 897* (in herb. Berol.) (holotype: B! [B10 0242218]; isotype: K! [K000308887]).

In his species description, Nilsson (1892) cited the collection *Schomburgk 897*, originating from British Guiana, undated, and deposited in the B herbarium. The unicate of this collection in B (B10 0242218) was examined, and is compatible with the protologue. Smith and Downs (1968) cited this sheet (B10 0242218) as “type”, thus identifying it as the holotype, which has two plants with the typical characteristics of *Xyris seubertii*, and the label indicates the location and the date (October 1842), as quoted in the protologue. Illustrations attached to the holotype sheet show characteristic features of the species, with a drawing of the central placenta and sepals with a thickly pilose carina and acute apex. However, in addition to these illustrations, there is an annotation signed by Seubert that indicates the species as *Xyris fontanesiana* Kunth, now considered as a synonym of *Xyris anceps* Lam. (Xyris section Xyris), which has a marginal placenta and is very different from *X. seubertii* (Xyris section Nematopus). An isotype is deposited at K (K000308887), with an annotation giving its origin from Guiana, and with indications of having previously been separated from another specimen. Another sheet at K (K000308888), also of *Xyris seubertii*, has the locality information “Roraima Expedition, British Guiana, Monte Roraima”. The collector is not mentioned, but the number 257 given on the label makes clear that it does not belong to the type collection.

### 13. Xyris simulans

Taxon classificationPlantaePoalesXyridaceae

L.A.Nilsson, Kongl. Svenska Vetenskap.-Akad. Handl. 24(14): 47. 1892.

#### Type.

“Brasilien”, Minas Gerais, Caldas. *Regnell III 1276* (lectotype, designated by Smith & Downs, 1968: S! [S 05-5679]; isolectotypes: UPS!; US [US00433375, image!]).

= ***Xyris tortula*** Mart. Flora 24(Beibl. 2): 55. 1841.

The lectotype of the *Regnell III 1276* (S 05-5679) material, designated as “type” by Smith and Downs (1968), is confirmed. Isolectotypes are deposited at US (US00433375) and UPS.

### 14. Xyris stenophylla

Taxon classificationPlantaePoalesXyridaceae

L.A.Nilsson, Kongl. Svenska Vetenskap.-Akad. Handl. 24(14): 46. 1892.

#### Type.

Brasil. *Glaziou 7999* (in herb. Berol.) (holotype: B! [B100242212]; isotypes: S! [S-R-6620], K! [K00494720]; P! [P00753728, P00753729]).

The holotype (B100242212) is the single sheet of *Glaziou 7999* at the B herbarium. It is from Brazil, undated, lacks a more precise location, and was indicated in the protologue as deposited at the B herbarium. The holotype consists of several plants and dried material that was used by Nilsson (1892) to describe the species. Smith and Downs (1968) cited the collection *Glaziou 7999* at the Stockholm herbarium (S R-6620) as “type”, but because Nilsson (1892) cited the B collection, the only sheet of this collection there, the S sheet must be considered as an isotype. Other isotypes were examined for the present study at K (K00494720) and P (P00753728, P00753729), all of *Glaziou 7999*, despite having annotations which give differing locations. The exsiccate K00494720 indicates that it is from Rio de Janeiro and P00753728 has a different indication to São Paulo, Campos Bocaina. However, the sheet P00753729 and the holotype (B100242212) were labeled only to Brazil, without mention of locality.

These disagreements in location attribution are thought to have occurred when Glaziou’s duplicates were distributed to the different herbaria.

### 15. Xyris teres

Taxon classificationPlantaePoalesXyridaceae

L.A.Nilsson, Kongl. Svenska Vetenskap.-Akad. Handl. 24(14): 44-45. 1892.

#### Type.

“Brasilien”. *Glaziou 4286* (lectotype, designated by Smith & Downs, 1968: P! [P00753726]; isolectotypes: K! [K00587113], US! [US00088220]). Other: *Glaziou 8003* (Itatiaia) (syntype: K! [K000587112]; isosyntype: P! [P01677114]); GH *n.v*.

[GH00028828]; *Sellow s.n.* (syntype: B! [B10 0242206]; isosyntypes: K! [K000837563, K000837566, K000837567]).

In the protologue, Nilsson cited three collections he had examined: *Glaziou 4286*, *Glaziou 8003* and *Sellow s.n.*, indicating the B herbarium as depository, however only the Sellow collection was found at B. Smith and Downs (1968) cited as “type” the material of *Glaziou 4286* deposited at P (P00753726), which is thus the lectotype, since there is no reason to disagree with the intention of these authors, the specimen being representative of the species and in agreement with the protologue. The lectotype consists of only a single plant, although a line drawn on the sheet shows that other, presumably mixed material has been removed; the label states that it is from the Serra dos Órgãos in Brazil, dated April 4, 1870. The duplicates at K (K000587113) and US (US00088220) are isolectotypes. The Kew isolectotype (K000587113) sheet, comprising young plants, also bears traces of previous mixtures, while the US isolectotype (US00088220) is mixed with *X. macrocephala* Vahl.

Duplicates of the syntype *Glaziou 8003* are deposited at K (K000587112) and P (P01677114), with annotations giving different origins. The P duplicate (P0167114), states its origin as Brazil, São Paulo, Fazenda Bocaina, near Cascatinha Manbucabo, collected on February 11, 1876. The K duplicate (K000587112) label annotation states the collection is from Rio de Janeiro, while another duplicate at GH (GH00028828) has data identical to the P specimen (Fazenda Bocaina, São Paulo). Despite this divergent locality information, there is no doubt that they all belong to the same Glaziou 8003 collection.

The duplicates of the syntype *Sellow* s.n. (B100242206, K000837563, K000837563, K000837566, K7000837567) indicate their origin only as from Brazil, with all bearing the number 204 on the label, confirming that all belong to the same *Sellow* collection. The collections examined revealed leaves with dark edges that can vary from bright to opaque, smooth to rugulose, variations observed in recent specimens of this species. The globular or ovoid spikes with brown bracts having a conspicuous macula are also very variable, sometimes making it difficult to identify this species, which overlaps in some characters with other similar species such as *Xyris rigida* Kunth.

## Supplementary Material

XML Treatment for Xyris cristata

XML Treatment for Xyris filifolia

XML Treatment for Xyris fusca

XML Treatment for Xyris glandacea

XML Treatment for Xyris glaziowii

XML Treatment for Xyris insignis

XML Treatment for Xyris laevigata

XML Treatment for Xyris longiscapa

XML Treatment for Xyris nigricans

XML Treatment for Xyris obtusiuscula

XML Treatment for Xyris regnellii

XML Treatment for Xyris seubertii

XML Treatment for Xyris simulans

XML Treatment for Xyris stenophylla

XML Treatment for Xyris teres
